# Liquid biopsy in oncology: a consensus statement of the Spanish Society of Pathology and the Spanish Society of Medical Oncology

**DOI:** 10.1007/s12094-019-02211-x

**Published:** 2019-09-26

**Authors:** J. Remon, R. García-Campelo, E. de Álava, R. Vera, J. L. Rodríguez-Peralto, Á. Rodríguez-Lescure, B. Bellosillo, P. Garrido, F. Rojo, R. Álvarez-Alegret

**Affiliations:** 1 Department of Medical Oncology, Centro Integral Oncológico Clara Campal Barcelona (CIOCCB), HM Delfos, Barcelona, Spain; 2 Department of Medical Oncology, Complexo Hospitalario Universitario A Coruña, A Coruña, Spain; 3 Institute of Biomedicine of Sevilla (IBiS), Department of Normal and Pathological Cytology and Histology, Virgen del Rocio University Hospital /CSIC/University of Sevilla, Sevilla, Spain; 4 CIBERONC, Madrid, Spain; 5 Department of Medical Oncology, Complejo Hospitalario de Navarra and Navarra Institute for Health Research (IdiSNA), Pamplona, Spain; 6 Department of Pathology, Hospital Universitario Doce de Octubre, Madrid, Spain; 7 Department of Medical Oncology, Hospital General Universitario de Elche y Vega Baja, Elche, Spain; 8 Group for Breast Cancer Research (GEICAM), Madrid, Spain; 9 Department of Pathology, Hospital del Mar, Barcelona, Spain; 10 School of Medicine, Universidad de Alcalá, Madrid, Spain; 11 Medical Oncology Department, IRYCIS, Hospital Universitario Ramón y Cajal, Madrid, Spain; 12 Department of Pathology, Fundación Universitaria Jiménez Díaz, Madrid, Spain; 13 Department of Pathology, Hospital Universitario Miguel Servet, Zaragoza, Spain

**Keywords:** Liquid biopsy, ctDNA, Oncology, Genomic profiling, Precision medicine

## Abstract

The proportion of cancer patients with tumours that harbour a potentially targetable genomic alteration is growing considerably. The diagnosis of these genomic alterations can lead to tailored treatment at the onset of disease or on progression and to obtaining additional predictive information on immunotherapy efficacy. However, in up to 25% of cases, the initial tissue biopsy is inadequate for precision oncology and, in many cases, tumour genomic profiling at progression is not possible due to technical limitations of obtaining new tumour tissue specimens. Efficient diagnostic alternatives are therefore required for molecular stratification, which includes liquid biopsy. This technique enables the evaluation of the tumour genomic profile dynamically and captures intra-patient genomic heterogeneity as well. To date, there are several diagnostic techniques available for use in liquid biopsy, each one of them with different precision and performance levels. The objective of this consensus statement of the Spanish Society of Pathology and the Spanish Society of Medical Oncology is to evaluate the viability and effectiveness of the different methodological approaches in liquid biopsy in cancer patients and the potential application of this method to current clinical practice. The experts contributing to this consensus statement agree that, according to current evidence, liquid biopsy is an acceptable alternative to tumour tissue biopsy for the study of biomarkers in various clinical settings. It is therefore important to standardise pre-analytical and analytical procedures, to ensure reproducibility and generate structured and accessible clinical reports. It is essential to appoint multidisciplinary tumour molecular boards to oversee these processes and to enable the most suitable therapeutic decisions for each patient according to the genomic profile.

## Introduction

By the year 2030, 22.2 million new cases of cancer are expected worldwide: a challenge for cancer patient diagnosis and therapeutic approaches. Despite this increase, patient prognosis has improved with a gradual decrease in cancer-related mortality [1, 2], reflecting the breakthroughs in early diagnosis and cancer therapy. Therapeutic advances are mainly based on the understanding that cancer is a heterogeneous genomic disease [3]. This has boosted the development of new tailored or precision therapeutic approaches that have a positive impact on patient survival.

The proportion of cancer patients with tumours harbouring potentially targetable genomic abnormalities at the start of treatment or during progression has been growing over time. This is the basis for precision medicine, crucial for taking therapeutic decisions and for understanding the therapy-induced dynamic evolution of the tumour [4]. At present, its use is considered standard in daily clinical practice for the treatment of some tumours [5], because it improves the outcome [6]. At the same time, drug approvals based on molecular abnormalities, regardless of the histology, have been enabled by precision oncology (tumour type-agnostic therapy approvals) [7]. Precision oncology has also helped to obtain information about predictive biomarkers, such as the tumour mutational burden (TMB), related to the efficacy of immune checkpoint inhibitors (ICI) in many cancer types [8].

This tailored treatment approach demands highly sensitive and precise technologies for molecular stratification [9]. However, it is not possible to determine the molecular profile in up to 25% of tumour biopsies, because the available tumour specimens do not meet the quality control criteria and have insufficient DNA for testing [9, 10]. Furthermore, biopsies provide limited information on the dynamics of tumour heterogeneity, as they can rarely be repeated sequentially because of their location, the tumour size and the risk of complications related to the procedure.

**Fig. 1:**
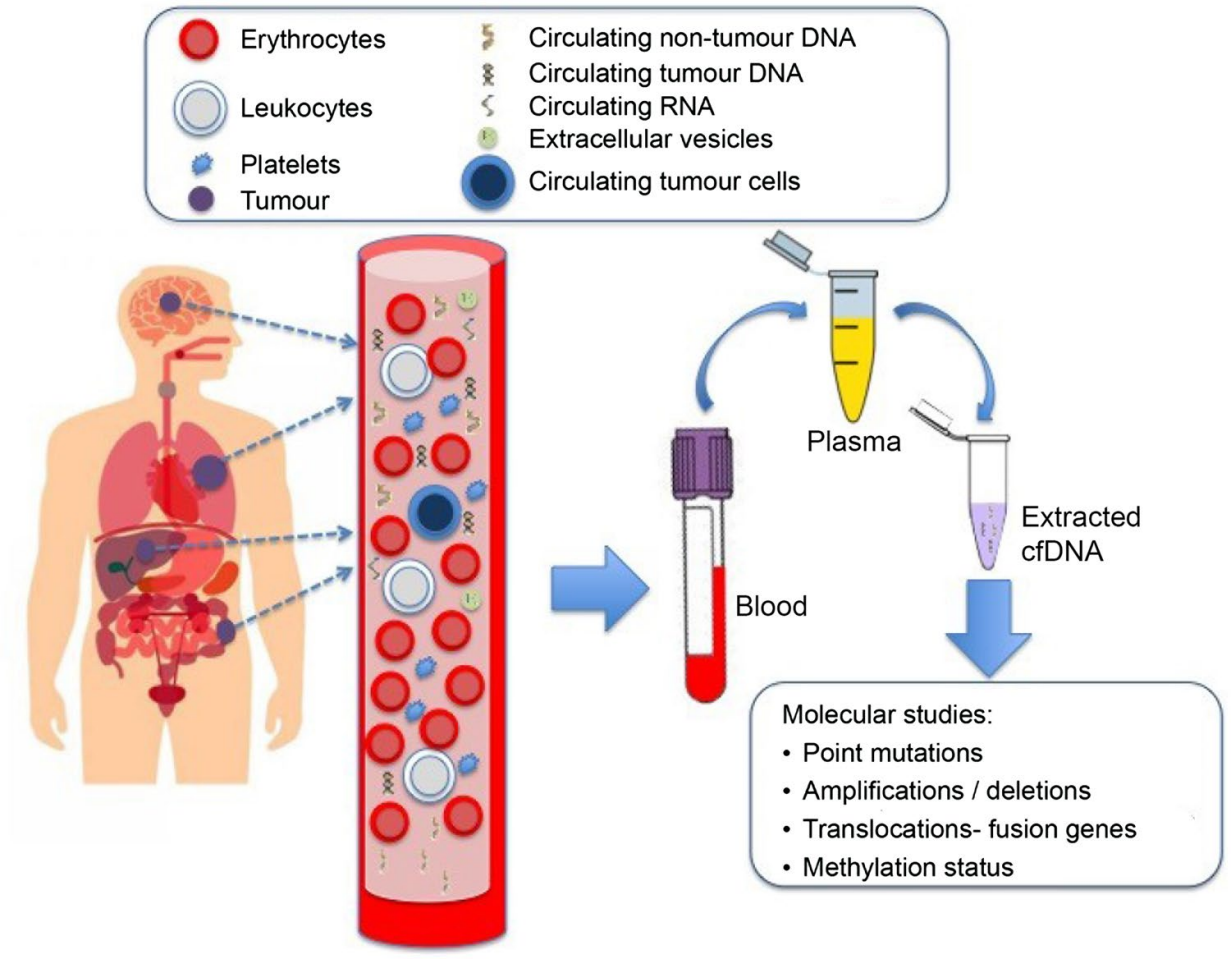
Graphic description of the process during liquid biopsy

Liquid biopsy, a term coined by Pantel and Alix-Panabières [11], is a non-invasive diagnostic technique that can establish tumour molecular profile at the start of treatment and during progression, and can also capture dynamic intra-patient genomic heterogeneity. Liquid biopsy includes testing for circulating tumour DNA (ctDNA), circulating tumour cells or exosomes, platelet RNA and circulating tumour RNA (ctRNA) in different fluids such as plasma, pleural fluid, urine or cerebrospinal fluid, among others, although blood is the most commonly used [12], as described in Fig. 1. However, the results of the different analytical techniques, including the most novel ones, have shown different levels of precision and performance in liquid biopsy [13].

One of the best developed forms of liquid biopsy in clinical practice is that of ctDNA testing for different tumours. This consensus statement will therefore be focused on the clinical value of ctDNA testing.

The objective of this consensus statement is to provide a joint vision from the Spanish Society of Pathology (SEAP) and the Spanish Society of Medical Oncology (SEOM) on the challenges and possibilities associated with ctDNA testing in cancer patients and to present physicians with precise and necessary information for decision making in daily clinical practice.

## Preanalytical requirements

### Specimen types

Peripheral blood and, more precisely, plasma is the most widely used specimen in liquid biopsy, mainly because it is easy to obtain and to manage. ctDNA constitutes a minor fraction of circulating free DNA (cfDNA) and it contains tumour-specific genomic abnormalities. The variant allele frequency (VAF) is the percentage of each specific mutation detected in cfDNA. The VAF can be very low in liquid biopsy, and it is therefore important to optimise preanalytical techniques to avoid false negatives.

The use of plasma sampling is preferred over serum for various reasons, even though the latter also contains ctDNA, such as the risk of cfDNA contamination from leukocyte lysis [14]. Furthermore, the ctDNA in serum samples can be partially adhered to a blood clot. Additionally, the platelet component may be lost in blood samples and this can be an important source of tumour nucleic acids.

### Collection

To obtain plasma, peripheral blood can be collected by venepuncture into tubes containing anticoagulant. The most widely used anticoagulant is ethylenediaminetetraacetic acid (EDTA), which inhibits DNase activity in the blood and is compatible with the polymerase chain reaction (PCR) test [15, 16]. The blood volume to be collected ranges from 15 to 20 ml. However, smaller volumes can be used for testing, thanks to technological advances.

There are specific blood collecting tubes for stabilising the sample and optimising plasma collection. These tubes prevent the lysis of blood cells and contamination by non-tumoral DNA for up to 1 week at a temperature of 22 °C [17]. However, some of these tubes contain ten times more additives than others (2.0 ml vs 0.2 ml), and this fact should be considered when calculating DNA yield [17].

### Management of samples

It is important that once the blood is drawn, it is processed within the first few hours after collection, 4–6 h at most, if EDTA tubes are used. Holding the samples at room temperature can cause massive lysis of blood cells, resulting in contamination with cfDNA [17]. This lysis is very obvious at 24 h, when the specimen has been stored at 22–24 °C [18–20]. Although some authors suggest that samples collected in EDTA tubes could be stable for up to 24 h [21], the general recommendation is that they should be processed within the first 6 h after collection [19, 20].

In case that immediate processing is not possible, tubes with a cell stabiliser should be used to preserve the integrity of blood cells present in the sample. This will prevent cell death, rupture and/or genetic material release, as the latter can dilute the ctDNA and hinder testing.

Peripheral blood sample processing consists of centrifugation for 10 min at 2800*g* and plasma separation followed by a second centrifugation at 16,000*g* for 10 min to ensure that all cell residue is removed. The plasma is then transferred to 1.5–2.0 ml tubes, which preferably have low nucleic acid binding capacity. It is advisable to divide the plasma into small volume aliquots (1–2 ml each), to take only the necessary volume when used and without needing to thaw all the available material, since cycles of freezing–thawing affect nucleic acid integrity.

### Storage and maintenance

According to published studies, storing frozen plasma before DNA extraction has no effect on subsequent ctDNA testing. For this reason, once the plasma is divided into aliquots, it should be stored frozen at – 20 °C for no more than 3 months or at – 80 °C if for longer periods (more than 3 months with no maximum period specification) [18].

## Testing methods

### Real time PCR (rtPCR) or quantitative PCR (qPCR)

RT-PCR or qPCR is a simple, quick and economic method for the relative quantification of somatic mutations, when compared against a control. The genomic alterations present in at least 10% of ctDNA can be detected with this technique [22]. Different types of qPCR have been developed to improve sensitivity: AS-PCR [23], AS-NEPB-PCR [24], PNA-LNA PCR clamp [25, 26] and COLD-PCR [27]. Most of these qPCR types are based on using an oligonucleotide that binds to the 3′ end of DNA to block amplification of the non-mutated allele and to promote amplification of the mutated allele. Alternatively, a step can be introduced in qPCR to enrich mutant allelic variants and facilitate their detection. AS-PCR is frequently used in clinical practice to detect single nucleotide variants or small insertions/deletions in paraffin-fixed tissue. However, although it has 98% specificity and 92% sensitivity with 96% concordance with the mutant allele in ctDNA samples [23], it is not the most adequate qPCR type for liquid biopsy. PNA-LNA PCR clamp has higher specificity, with a 0.1% detection limit of the mutant allele and 79% specificity [25, 26]. However, the most robust qPCR type for mutant variant detection is COLD-PCR, with a 0.1% detection limit and an enrichment of the mutant allele to improve the detection sensitivity of the technique up to 100 times [27].

### Digital PCR (dPCR)

dPCR is a method that is more sensitive than qPCR. The sensitivity of this technique for mutant detection is close to 0.1% and it is also a relatively economical, quick and simple method for absolute quantification of somatic mutations present in ctDNA [28]. The high sensitivity and specificity of dPCR means that it is an especially useful technique for liquid biopsy. dPCR consists of distributing DNA from the specimen into thousands or millions of partitions made in oil droplets generated with a water–oil emulsion (digital droplet PCR [ddPCR]) [28, 29] or in multiple wells in a physical support [30]. Each partition contains one fragment of single-chain DNA, mutant or non-mutant, which will be clonally amplified by PCR. Mutant and non-mutant DNA is detected using fluorescent TaqMan® probes that can detect and quantify mutations that are very uncommon, but relevant in the tumour [31–33].

### BEAMing

Liquid biopsy using BEAMing technology (Beads, Emulsification, Amplification and Magnetics) is a system for non-invasive study of tumour genotype based on the presence of ctDNA in peripheral blood. It is therefore possible to evaluate the presence of mutations with prognostic or predictive value and also to quantify them.

After isolating the DNA, the regions of interest are amplified by PCR. Amplified DNA sequences are bound to magnetic beads impregnated with specific oligonucleotides and divided in millions of aqueous microdroplets in a water–oil emulsion, so that each microdroplet will contain only one DNA molecule and one magnetic particle. After subjecting the microdroplets to temperature cycles similar to conventional PCR, each sequence is amplified again using the oligonucleotides as primers. After, the beads that are bound to thousands of DNA copies having the sequence of interest are collected.

Once this process has been completed, the aqueous phase is separated from the oily phase and the magnetic microbeads are collected, purified and stained with specific fluorophores to identify the mutant and non-mutant sequences due to their different fluorescence. Finally, the proportion of beads with mutant DNA compared with the control is determined by conventional flow cytometry [34, 35].

### NGS

Next generation sequencing (NGS) technology allows parallel sequencing of millions of small DNA fragments. The sequences are then integrated using bioinformatics tools to detail the sequences of large genetic structures quickly, precisely and economically. Known mutations, as well as new mutations, fusions, abnormal gene copy number, mutational burden or microsatellite instability can be detected by applying NGS techniques [36]. NGS-based liquid biopsy, unlike tissue NGS, requires a high sequencing depth, as well as incorporating molecular barcoding to differentiate errors in sequencing from real mutations and to achieve high sensitivity.

There are several approaches to NGS, the most important being sequencing by ligation and sequencing by synthesis. In the first case, the DNA sequence is obtained from the fluorescence emitted after hybridisation with fluorophore-labelled probes (such as the *Illumina®* platform). DNA fragmentation and amplification is necessary before sequencing. In the case of *Illumina*^*®*^, sequencing is performed by PCR on a solid support. Hybridisation and the subsequent complementary chain synthesis by DNA polymerase take place in a flow cell coated with two types of primers, one of them complementary to the DNA sequence. DNA is denatured and the final domain of the amplified fragment is bound to the second type of primer, creating a bridge that acts as a pattern that repeats the process thousands of times.

In the case of *Ion Torrent*^*®*^*,* changes in pH or fluorescence are produced, when new nucleotides are incorporated by polymerases and these are translated into the sequence. Amplification takes place in an emulsion, where DNA is bound to the specific primers inside microdroplets, generating thousands of sequences in each emulsion.

With regards to the sequencing step, cyclic reversible termination incorporates four fluorophore-labelled nucleotides that block the amplification process and are excited by a light source, with an intensity and wavelength that determine the synthesis sequence. The difference lies in the possibility of reversing the blockage, recovering the 3′-OH end, and therefore being able to continue adding nucleotides, to break the chemical bonds and eliminate the fluorophore attached to the nucleotide. This process takes place on large scale and in parallel in the cell.

In sequencing by synthesis by the addition of a single nucleotide or pyrosequencing, nucleotides are added in sequence. If the DNA polymerase incorporates the nucleotide to extend the primer and afterwards pauses, an inorganic pyrophosphate is released that is then transformed in visible light by a series of enzymmatic reactions. A sensor detects the signal and depicts it in a pictogram, which allows the sequence to be determined. Before adding the next nucleotide, an apyrase degrades the excess nucleotides from the last step to avoid inaccurate reactions [37, 38].

Table 1 shows the main advantages and disadvantages of testing methods that can be used in liquid biopsy.

**Table 1:** Advantages and disadvantages of testing methods that can be used in liquid biopsy

Technology	Advantages	Disadvantages
rtPCR or qPCR	Quick	Low sensitivity
	Simple	Low specificity
	Economical	Detects already known mutations
ddPCR	High sensitivity	Limited to multiplexing variant detection in a single reaction
	High specificity	Detects already known mutations
	Quick	
	Simple	
	Relatively economical	
BEAMing	Quick	Lacks validation?
	Slightly invasive	
	Simple	
	Relatively economical	
NGS	High precision	Complicated preparation of specimens
	High reproducibility	Limited to certain DNA regions
	Detects new mutations	Requires a complex bioinformatic analysis
	Price progressively becoming lower	

*BEAMing* beads, emulsification, amplification and magnetics, *ddPCR* droplet-digital polymerase chain reaction, *NGS* next generation sequencing, *qPCR* quantitative PCR, *rtPCR* real time PCR

## Clinical validity and utility

The validity of liquid biopsy (measured as the capacity of a test to divide a population into groups with significantly different clinical results) and the clinical utility (measured as the capacity of a test to improve cancer diagnosis, treatment, management or prevention results) are the objectives of current oncology studies on liquid biopsy [39, 40].

### Early cancer detection

To date, liquid biopsy is not considered a sufficiently sensitive or specific technique for early cancer detection in an asymptomatic population and cannot substitute for or complement radiological tests. Despite this the potential of liquid biopsy in this scenario is increasingly evident due to current technological advances. The exploratory validation of this technique is essential, as is the standardisation of preanalytical processes. To interpret the results correctly, it is important to consider the detection levels of the method to avoid false positives and to discern abnormalities that have no oncogenic potential. It is also important that studies are conducted to compare case and control populations with an optimal population number.

Recently, a study conducted by the UK’s Early Cancer Detection Consortium (ECDC) has evidenced the need to standardise sample size, design and testing procedures in liquid biopsy studies before incorporating such strategies into screening programs [41].

However, most recent studies have shown that there is evidence of the potential utility of liquid biopsy in early cancer detection [42]. One example of this is the results of the Circulating Cell-free Genome Atlas (CCGA) study [43]. CCGA is a prospective cohort study designed for early cancer detection that will include 15,000 participants; 70% with a cancer diagnosis and 30% healthy participants, with no restrictions on comorbidities. In a planned case–control analysis with 2800 participants split into two groups: training group with *n* = 1406 (845 with different cancers, including 118 lung cancer patients; and 561 non-cancer patients) and the independent group enrolling *n* = 834 (*n* = 472 cancer patients, including 46 lung cancer patients; and 362 non-cancer patients), three methodologies for cfDNA detection have been used: (1) targeted sequencing of somatic mutations; (2) whole genome sequencing (WGS) and; (3) whole genome bisulphite sequencing (WGBS) These three methods yield similar results. WGBS detected 41% of stage I, II, IIIA lung tumours and 89% of advanced stage tumours (IIIB–IV). WGS detected 30% of early stage tumours and 87% of advanced tumours and targeted sequencing detected 51% of early stage tumours and 89% of advanced tumours, showing that using cfDNA for lung cancer screening is a promising technique with a very low rate of false positives (< 1%).

The CancerSEEK panel has been developed for early detection in eight main cancers (ovaries, liver, pancreas, stomach, oesophagus, colorectal, breast and lung) and combines the evaluation of 16 genes in ctDNA with a sensitivity between 69 and 98% and a specificity higher than 99% [42]. The role of liquid biopsy as a useful tool for early cancer diagnosis, alone or combined with other techniques, will be established by prospective validation with techniques that are standardised and that have good preanalytical controls.

### Detecting residual disease in early disease

Early detection of tumour recurrences, after radical local treatment using dynamic ctDNA monitoring, poses a new challenge for early therapeutic decision making, which is, to date, based on clinical parameters and TNM staging. In localised disease, the proportion of detected ctDNA is lower than in advanced disease [44]. The persistence of ctDNA after radical treatment is correlated with the persistence of minimal residual disease (MRD) in many tumour types such as breast, lung or colon cancer. Detecting this type of ctDNA is correlated with a poorer prognosis and the diagnosis of relapse can be established before standard radiological procedures with high sensitivity and specificity [45–48]. If MRD could be detected through ctDNA, the population eligible for adjuvant therapy could be better defined.

The challenges in the development of future studies will include important methodological aspects that are not yet clarified, such as whether ctDNA should be measured using a binary variable (positive/negative) or a continuous one; or the standardisation of the data obtained from several studies, employing different techniques [49].

### Molecular profile in advanced disease

The clinical validity of ctDNA testing using qPCR to detect *EGFR* mutations in non-small-cell lung cancer (NSCLC) and *KRAS* mutations in colorectal cancer (CCR) has been proven [50–52] and it has the approval of the FDA and the EMA.

Previously untreated advanced NSCLC is one of the main settings where ctDNA testing can be used in molecular profile analysis and, based on the results, for therapeutic decision making in clinical healthcare practice. The criteria for selecting the population eligible for molecular testing using ctDNA at diagnosis are the same ones as those recommended for tissue testing and they are gathered in the main national and international clinical guidelines. These criteria are: patients with advanced non-squamous NSCLC and squamous NSCLC with certain clinical characteristics (non-smokers, young patients…) that could be indicative of a potentially targetable genetic abnormality; when there is no tumour specimen available or one with low cellularity; or with inaccessible lesions for diagnosis or not worthwhile (bone lesions) as they require a dangerous procedure for obtaining the tumour specimen [53]. A negative ctDNA test result using a validated methodology should be confirmed in tumour tissue. Given the limited evidence of the clinical validity, beyond the significance of ctDNA for studying mutations in *EGFR* in NSCLC and *KRAS* in CRC, and given the number of potentially targetable genetic alterations, there is a growing interest in using new strategies, such as NGS panels, although the experience is limited [54]. However, early studies show a good agreement with the genomic alterations obtained with tissue, although this agreement can be compromised by the variants detected in ctDNA with a VAF < 1% [55].

### Diseases monitoring

ctDNA monitoring seems to be a possible alternative to imaging techniques. Changes in ctDNA levels can predict tumour progression with a difference of several months compared with conventional methodology in some tumours [56]. The main applications of this technique include response monitoring, a better definition of questionable stable disease or disassociated response and even early response assessment entailing a treatment change, without having to wait for weeks for radiological evaluation. There are various studies with limited numbers of patients conducted in different tumour types, as well as many retrospective studies, that have shown a good correlation between changes in ctDNA and response [56–58]. To implement ctDNA quantification in daily clinical practice, further studies must be conducted to prove the efficacy and reproducibility of the methodology used and also the impact at a clinical level of the modified therapeutic approach based on biological progression (according to ctDNA levels) with respect to conventional radiological progression. To date, there is insufficient evidence to recommend using liquid biopsy for disease monitoring or for therapeutic decision making based on this methodology.

### Detecting resistance mechanisms

Many studies have been published that demonstrate that ctDNA can be used for emergency monitoring of resistant clones during exposure to a predetermined targeted treatment strategy such as *T790M* mutation following treatment with *EGFR* tyrosine kinase inhibitors (TKIs), resistance mutations to *ALK* in patients carrying the translocation after exposure to *ALK* inhibitors [59], *ESR1* mutations [60, 61]*, PIK3CA* in breast cancer patients treated with different treatment strategies, or *KRAS* mutations in colorectal carcinoma patients treated with anti-EGFR drugs [62–64]. Once again, a good example of how detecting one of the most common mechanisms of resistance (*T790M*) to first-generation or second-generation inhibitor drugs has been implemented in the context of clinical practice is advanced NSCLC with *EGFR* mutations, detected in ctDNA using Cobas® with a moderate sensitivity. This method has been recommended in national and international guidelines [53, 65]. Nevertheless, there are other techniques with greater sensitivity that can be adequate for detecting this acquired mutation.

An adequate and advisable technique is the use of NGS panels, since they increase the possibility of detecting resistance mechanisms other than *T790M*, and therefore the treatment can be tailored during progression. This is important, as third-generation inhibitors such as osimertinib are therapeutic options in first-line therapy for *EGFR*-mutant patients [66]. Using liquid biopsy, *MET* amplifications (15%) and *EGFR* mutations such as *C797S* (7%) have been reported as the major resistance mechanisms to osimertinib and this finding can enable clinical trials with targeted therapies according to the genomic profile during progression using NGS [67].

### The role of liquid biopsy in immunotherapy

Liquid biopsy is a novel and promising research field in the search for predictive and prognostic biomarkers in immunotherapy.

ctDNA levels can have a prognostic and predictive value in patients with advanced tumours treated with immunotherapy. In a small study of 19 metastatic melanoma patients treated with anti-CTLA-4 or anti-PD-1, ctDNA levels at baseline ≥ 10 copies/ml, were associated with a poorer prognosis than ctDNA levels < 10 copies/ml (HR 6.3; p 0.017) [68]. In another study of advanced melanoma, patients treated with ICI, ctDNA levels detected at the start were associated with worse results in terms of progression free-survival (PFS) and overall survival (OS) in a univariate analysis [69]. Monitoring ctDNA levels could play a role in response monitoring and also in a current very uncertain clinical setting: detecting and differentiating pseudoprogression from true progression or even identifying hyperprogressors [68, 70, 71]. Another application in the future could be detecting resistance mechanisms to ICI, such mutations in *JAK2*, *CTNNB1*, *BRCA2*, *PTEN* or *B2M*, that have been previously described as potential resistance mechanisms to several tumours treated with ICI and that can be detected in ctDNA [72].

With regard to predictive markers, a high TMB can increase the appearance of neoantigens, thereby enhancing immunotherapy response. In fact, measuring TMB in tumour tissue has shown a predictive potential in several tumour types [73, 74]. At the same time, a significant effort has been devoted to measuring TMB retrospectively in peripheral blood (bTMB) in many tumour types and to estimate its predictive value. Recently, bTMB data from two prospective studies have been published. The studies were conducted in previously treated advanced NSCLC patients and compared docetaxel with atezolizumab. In these studies, a high bTMB was correlated with a benefit in PFS with immunotherapy [75]. It is important to note that in the study, a high bTMB was not correlated with PD-L1 expression levels. In this regard, the results of the B-FIRST [76] study have recently been published. This study is the first prospective study evaluating bTMB as a predictive biomarker in advanced NSCLC patients treated with atezolizumab monotherapy as first-line therapy. The results of this analysis have shown that greatest benefits in PFS are seen in patients with high bTMB and have better responses with atezolizumab versus chemotherapy, but not in OS [76]. Considering this data, the consensus is that, to date, there is not enough evidence to recommend the use of liquid biopsy in immunotherapy, since there is a lack of standardisation in the technique for detecting bTMB as well as in the cut-off points for defining high bTMB.

Table 2 shows a summary of the validity and clinical utility of liquid biopsy at different points in the disease course.

**Table 2:** Validity and clinical utility of liquid biopsy in clinical practice

	Approval status
Screening	Not approved
Minimal residual disease	Not approved
Advanced disease	Approved for NSCLC and CRC
Disease monitoring	Not approved
Resistance mechanisms	Approved for *T790M* in NSCLC
Immunotherapy	Not approved

*CRC* colorectal cancer, *NSCLC* non-small cell lung cancer

## Interpreting the results

ctDNA coexists with cfDNA. The feasibility of liquid biopsy depends on the amount of detected ctDNA, although different factors such as the amount and site of metastases (except in patients with metastatic brain disease), the cell proliferation index, the apoptosis rate, the genomic instability or the amplification of a gene associated with a mutation can be limiting factors [45, 77]. These limitations could explain the differences between the results obtained in liquid biopsy and those obtained with tissue [44]. Therefore, it should be borne in mind that a negative liquid biopsy test does not necessarily mean an absence of a genomic abnormality.

The growing application of quantitative techniques, such as NGS or the different versions of dPCR, has reduced the limitations mentioned above. These techniques can detect mutations and quantify a mutation frequency using VAF. Sequential evolution in the VAF variants of a patient’s genomic alterations can be considered as a longitudinal marker to replace tumour evolution or therapeutic response. Tumour heterogeneity can be detected with liquid biopsy [78] and with the VAF of the differently detected abnormalities and the coexistence of dominant clones, indicative of the responsiveness to a targeted therapy can be established [79, 80] as well as the coexistence of sub-clonal alterations with uncertain significance or that can be associated with a poorer prognostic [81].

The portfolio of targeted therapies includes the treatments approved for specific molecular alterations as well as experimental drugs that have limited preclinical evidence [82]. As NGS techniques provide more information, interpreting and prioritising clinically relevant genomic alterations poses a significant challenge. Another critical aspect in precision oncology is defining standardised bioinformatics procedures and developing algorithms that determine which genetic alterations should guide the selection of a targeted therapy [83]. It is therefore crucial and a real challenge to create multidisciplinary tumour molecular boards focused on genomic profiling tests for tumours. These boards will help providing objective interpretations of results that follow any of the current classifications or consensus statements based on scientific evidence [84] and that make a real impact in therapeutic decision making. For this purpose, it is important that the results reported from the ctDNA test are precise and clear so that the necessary information for therapeutic decision making is transmitted. Apart from patient and sample identification data, the report should include the details regarding the method used, the analytical characteristics of the assay, the sensitivity or detection limit of the mutated allele and the VAF, if available in that assay [85, 86].

Likewise, there are still important technical and ethical barriers that should be evaluated before implementing NGS in clinical practice. In this respect, the same genomic alteration can have distinct therapeutic implications in different tumour contexts [87]. Furthermore, not all somatic variants detected in plasma are derived from the tumour. Some mutations can be related to clonal haematopoiesis processes [88, 89] that are more frequent from the fifth decade of life, occurring in up to 10% of healthy individuals 70 years of age or older [90–92]. ctDNA testing using NGS can detect both somatic and germline variants, the latter characterised by a VAF were higher than 50%. Finally, the psychological impact on the patient should be considered, when non-targetable genomic alterations are detected.

## Other considerations

### Informed consent

The informed consent form should be precise, concise and accessible. Liquid biopsy is a rapid growth field in oncology and NGS techniques in ctDNA can provide a substantial amount of genomic information, including germline mutation detection in ctDNA in up to 1.4% of cases, especially in patients younger than 50-years old for all tumour types [93]. In this context, healthcare professionals can face an ethical dilemma, when revealing germline results detected in liquid biopsy that do not have a repercussion in practice [94], but that can have a psychological impact on the patients and their families [95]. The informed consent should include these considerations, and both the patients and their families should be advised about this. The patient should also express if he/she wants to know the result, in case a germline alteration was detected. Widespread use of NGS can increase incidental detection of germline mutations in cfDNA and it can become an important challenge in coming years, requiring collaboration from Genetic Committee units. However, it is worth noting that, to date, germline mutations detected in cfDNA should not replace validated genetic testing for hereditary cancer. Finally, the document should note that there is a possibility that conducting these tests does not detect an alteration or that the detected alterations could potentially not be targetable at present.

### Quality control

Quality control during the test phase should be conducted routinely to predict and prevent procedural failures and to detect possible false negatives.

The technical procedure (e.g. dPCR, NGS) should be validated to simulate the clinical environment. Furthermore, the assay sensitivity and specificity should be robust, reproducible and should have proper internal and external quality controls [13, 19, 96, 97]. Comparisons with paired tissue specimens should have the same characteristics. Some authors (89) suggest using synthetic controls that imitate the DNA in the patient’s plasma [98].

Taking part in external quality programs (EQA) is essential, both for the preanalytical phase already discussed as well as for the quantification and genotyping methodology. There are several providers or associations for validating the technique: European Molecular Genetics Quality Network (EMQN), European Society of Pathology (ESP), EQA and the United Kingdom National External Quality Assessment Service (UK NEQAS) for Molecular Genetics, sponsored by the International Network for Pathology (IQN Path) [96, 97].

## Conclusions

In some tumours, liquid biopsy is a valid alternative to current standard procedures, offering rapid, precise and dynamic information that can complement the information offered by a tumour biopsy. It can describe the heterogeneity of the tumour and it can also provide relevant information for therapeutic decision making at baseline and during progression. For this reason, in some tumours and according to current evidence, liquid biopsy is considered to be an acceptable alternative to tumour tissue biopsy.

To gain wide acceptance and increase implementation of liquid biopsy in routine practice by professionals that treat cancer patients, it is important to standardise preanalytical and analytical procedures, so they are reproducible and also to generate structured and accessible reports. Multidisciplinary tumour molecular boards focused on evaluating the genomic profile of the tumour are necessary for this process to validate and integrate the genomic profiling results in the clinical setting. The potential applications of ctDNA such as early diagnosis, screening or molecular residual disease detection are the challenges for the future, as they can increase the utility of these techniques in the early stages of cancer. Detecting the mechanism of acquired resistance to various tailored treatments is also a challenge in advanced disease. Therefore, improving our knowledge on the clinical utility of liquid biopsies will help to implement this technique in the broad management of cancer patients.
