# Correction to: Liquid biopsy in oncology: a consensus statement of the Spanish Society of Pathology and the Spanish Society of Medical Oncology

**DOI:** 10.1007/s12094-020-02313-x

**Published:** 2020-02-06

**Authors:** J. Remon, R. García-Campelo, E. de Álava, R. Vera, J. L. Rodríguez-Peralto, Á. Rodríguez-Lescure, B. Bellosillo, P. Garrido, F. Rojo, R. Álvarez-Alegret

**Affiliations:** 1 Department of Medical Oncology, Centro Integral Oncológico Clara Campal Barcelona (CIOCCB), HM Delfos, Barcelona, Spain; 2 Department of Medical Oncology, Complexo Hospitalario Universitario A Coruña, A Coruña, Spain; 3 Institute of Biomedicine of Sevilla (IBiS), Department of Normal and Pathological Cytology and Histology, Virgen del Rocio University Hospital /CSIC/University of Sevilla, Sevilla, Spain; 4 CIBERONC, Madrid, Spain; 5 Department of Medical Oncology, Complejo Hospitalario de Navarra and Navarra Institute for Health Research (IdiSNA), Pamplona, Spain; 6 Department of Pathology, Hospital Universitario Doce de Octubre, Madrid, Spain; 7 Department of Medical Oncology, Hospital General Universitario de Elche y Vega Baja, Elche, Spain; 8 Group for Breast Cancer Research (GEICAM), Madrid, Spain; 9 Department of Pathology, Hospital del Mar, Barcelona, Spain; 10 School of Medicine, Universidad de Alcalá, Madrid, Spain; 11 Medical Oncology Department, IRYCIS, Hospital Universitario Ramón y Cajal, Madrid, Spain; 12 Department of Pathology, Fundación Universitaria Jiménez Díaz, Madrid, Spain; 13 Department of Pathology, Hospital Universitario Miguel Servet, Zaragoza, Spain

## Correction to: Clinical and Translational Oncology 10.1007/s12094-019-02211-x

The article Liquid biopsy in oncology: a consensus statement of the Spanish Society of Pathology and the Spanish Society of Medical Oncology, written by J. Remon, R. García-Campelo, E. de Álava, R. Vera, J. L. Rodríguez-Peralto, Á. Rodríguez-Lescure, B. Bellosillo, P. Garrido, F. Rojo and R. Álvarez-Alegret was originally published electronically on the publisher’s internet portal on 26 September 2019 without open access.

With the author(s)’ decision to opt for Open Choice the copyright of the article changed on 6 February 2020 to © The Author(s) 2020 and the article is forthwith distributed under a Creative Commons Attribution 4.0 International License (https://creativecommons.org/licenses/by/4.0/), which permits use, sharing, adaptation, distribution and reproduction in any medium or format, as long as you give appropriate credit to the original author(s) and the source, provide a link to the Creative Commons licence, and indicate if changes were made.

The original article has been corrected.

